# Global research trends on ALK-TKIs in non-small cell lung cancer: a bibliometric analysis

**DOI:** 10.3389/fphar.2025.1665174

**Published:** 2025-08-15

**Authors:** Yafeng Liu, Zhaojin Han, Yujia Li, Pengfei Zhang, Qiushi Wang

**Affiliations:** Department of Thoracic Surgery, Second Affiliated Hospital of Harbin Medical University, Harbin, China

**Keywords:** NSCLC, ALK mutation, ALK-TKIs, bibliometric analysis, Citespace, VOSviewer

## Abstract

**Background:**

Lung cancer remains the leading cause of cancer-related incidence and mortality worldwide. Non-small cell lung cancer (NSCLC) constitutes the most prevalent histological subtype of lung cancer. A notable proportion of NSCLC patients harbor mutations in the anaplastic lymphoma kinase (ALK) gene, and treatment with ALK-TKIs has demonstrated favorable therapeutic efficacy in ALK-positive patients. This study aimed to systematically analyze the body of literature on ALK-TKIs in NSCLC over the past decade from a bibliometric perspective.

**Methods:**

Relevant literature on anaplastic ALK-TKIs for the treatment of NSCLC published between 2015 and 2024 was retrieved from the Web of Science Core Collection. Only English-language publications categorized as original researches and reviews were included. Additionally, clinical trial data from the past decade were collected from the ClinicalTrials.gov database. Bibliometric analysis, data processing, and visualization were conducted using CiteSpace, VOSviewer, Excel, and R.

**Results:**

Between 2015 and 2024, a total of 2,877 publications on ALK-TKIs for NSCLC were identified, with the annual output remaining consistently high, and 198 clinical trials were registered on ClinicalTrials.gov. China contributed the highest number of publications, while Massachusetts General Hospital emerged as the most prolific institution. The most influential journal in this field was *Lung Cancer*, and Alice T. Shaw was both the most prolific and one of the most influential authors. Keywords such as ‘lorlatinib’, ‘resistance’, ‘circulating tumor DNA’, and ‘immunotherapy’, along with keyword clustering, indicate current research hotspots and future directions in this field.

**Conclusion:**

This study provides a comprehensive bibliometric analysis and summary of the developmental trajectory, current research landscape, and future trends in ALK-TKI therapy for NSCLC over the past decade, with individualized and precision medicine remaining the primary direction for the development of ALK-TKI therapy in NSCLC.

## 1 Introduction

Globally, lung cancer remains the leading cause of cancer-related incidence and mortality, with approximately 2,480,000 new cases and 1,817,000 deaths annually (Bray et al., 2024). Non-small cell lung cancer (NSCLC) is the most prevalent histological subtype, accounting for approximately 85% of all lung cancer cases (Duma et al., 2019). Over the past 2 decades, rapid advancements in molecular targeted therapies have significantly improved the prognosis of NSCLC patients (Tan and Tan, 2022).

Anaplastic lymphoma kinase (ALK) represents a key therapeutic target in NSCLC, with ALK gene rearrangements first identified in 2007 (Soda et al., 2007). ALK gene fusions have been detected in approximately 3%–7% of NSCLC cases. The emergence of tyrosine kinase inhibitors (TKIs) targeting ALK rearrangements has demonstrated superior survival benefits compared to conventional chemotherapy, leading to improved prognoses for ALK-positive NSCLC patients (Qin et al., 2024). Anaplastic lymphoma kinase (ALK) is a critical therapeutic target in NSCLC. First identified in 2007, ALK gene rearrangements result from an inversion on the short arm of chromosome 2p, leading to the formation of fusion genes between the echinoderm microtubule-associated protein-like 4 (EML4) and ALK genes in NSCLC cells [4]. These fusions are present in approximately 3%–7% of NSCLC cases. ALK rearrangements activate oncogenic signaling pathways, including Janus kinase/signal transducer and activator of transcription (JAK-STAT), mitogen-activated protein kinase/extracellular signal-regulated kinase (MAPK/ERK), and phosphatidylinositol 3-kinase/protein kinase B (PI3K/AKT) pathways (Qin et al., 2024; Zou et al., 2007; Fumarola et al., 2014). Following the recognition of ALK rearrangements as key oncogenic drivers in NSCLC, crizotinib—an ALK-targeted tyrosine kinase inhibitor (TKI)—was developed. Crizotinib exerts its antitumor effects by inhibiting receptor tyrosine kinases along the ALK signaling axis. Compared with traditional chemotherapy, crizotinib significantly prolongs progression-free survival (PFS) and reduces treatment-related adverse events, thus improving both prognosis and quality of life in ALK-positive NSCLC patients (Solomon et al., 2014). Since the approval of the first-generation ALK-TKI crizotinib by the U.S. Food and Drug Administration (FDA) in 2011, the field has undergone rapid development (Shaw et al., 2011). Subsequent second- and third-generation ALK-TKIs have been developed to overcome the limitations of first-generation agents, including poor blood-brain barrier penetration and acquired drug resistance, and to target ALK-resistant mutations that emerge in some NSCLC patients during treatment. Second-generation ALK-TKIs such as alectinib have demonstrated significantly improved clinical outcomes compared to the first-generation ALK-TKI crizotinib, including higher event-free survival (EFS) and PFS rates, fewer central nervous system (CNS) metastases, and lower incidence of grade 3–5 adverse events (Peters et al., 2017). Similarly, the second-generation ALK-TKI brigatinib provides longer PFS and superior efficacy in patients with CNS involvement when compared to crizotinib (Camidge et al., 2021). To further enhance clinical efficacy, improve outcomes in patients with disease progression after second-generation ALK-TKI therapy, and increase control over CNS metastases, third-generation ALK-TKIs such as lorlatinib have been developed. Long-term follow-up data have shown that lorlatinib achieves a remarkably prolonged median PFS of over 60 months, compared to 9.3 months for crizotinib, and significantly reduces the risk of intracranial progression (Solomon et al., 2024). According to the NCCN guidelines, patients with ALK-rearranged NSCLC are recommended to receive first-line treatment with approved ALK-TKIs such as alectinib, brigatinib, or crizotinib. Upon disease progression, continued ALK-TKI treatment in combination with definitive local therapy may be considered depending on the clinical scenario. For patients who develop extensive metastatic disease and have not received lorlatinib previously, lorlatinib or systemic therapy is recommended. In patients progressing after crizotinib, second- or third-generation ALK-TKIs plus local therapy are recommended, except in asymptomatic cases where crizotinib may be continued. For patients with widespread metastases, subsequent options include second- or third-line ALK-TKIs such as alectinib, brigatinib, ceritinib, or lorlatinib, or systemic therapy as appropriate (Riely et al., 2024). Numerous novel agents are currently in the preclinical stage. Throughout the iterative development of ALK-TKIs for NSCLC, extensive studies have explored resistance mechanisms, novel biomarker identification, and combinatorial therapeutic strategies. These advances have resulted in a substantial body of literature encompassing preclinical studies, clinical trials, real-world evidence, and translational research.

Although numerous studies on ALK-TKIs in NSCLC have been published in recent years, systematic investigations into development trends and research hotspots remain limited. Bibliometric analysis is a quantitative method used to extract and analyze large volumes of scientific literature to map global collaboration networks, quantify research contributions, and identify emerging hotspots (Ninkov et al., 2022).

The aim of this study is to conduct a comprehensive bibliometric analysis of studies and clinical trials on ALK-TKIs in NSCLC over the past decade, in order to present an integrated overview of the developmental trajectory, current landscape, and emerging trends in ALK-TKI research for NSCLC treatment. This analysis may assist researchers in efficiently understanding the current state of the field and offer insights into future research directions.

## 2 Materials and methods

### 2.1 Data source and search strategy

**FIGURE 1:**
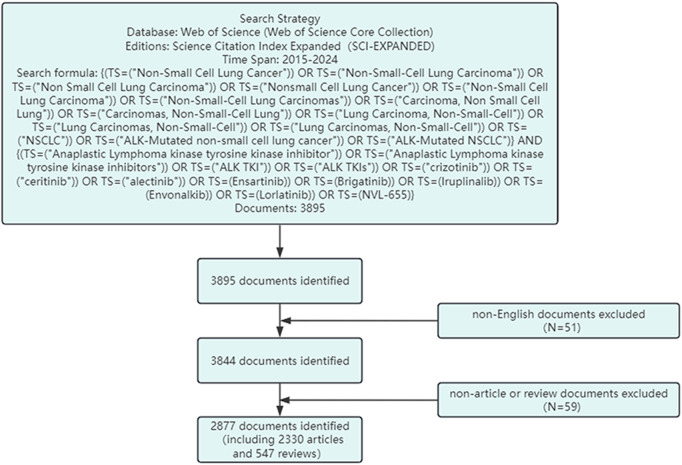
Search strategy and data selection process.

This study utilizes data primarily from the Web of Science Core Collection and the ClinicalTrials.gov database. The Web of Science provides comprehensive and standardized bibliometric data and includes the largest number of indexed journals, and is therefore widely used in bibliometric research (Wu et al., 2022; Xu et al., 2023). The search strategy used in the Web of Science database was as follows: (TS=(“Non-Small Cell Lung Cancer” OR “Non-Small-Cell Lung Carcinoma” OR “Non Small Cell Lung Carcinoma” OR “Nonsmall Cell Lung Cancer” OR “Non-Small Cell Lung Carcinoma” OR “Non-Small-Cell Lung Carcinomas” OR “Carcinoma, Non Small Cell Lung” OR “Carcinomas, Non-Small-Cell Lung” OR “Lung Carcinoma, Non-Small-Cell” OR″Lung Carcinomas, Non-Small-Cell” OR “Lung Carcinomas, Non-Small-Cell” OR “NSCLC” OR “ALK-Mutated non-small cell lung cancer” OR “ALK-Mutated NSCLC”)) AND (TS=(“Anaplastic Lymphoma kinase tyrosine kinase inhibitor” OR “Anaplastic Lymphoma kinase tyrosine kinase inhibitors” OR “ALK TKI” OR “ALK TKIs” OR Crizotinib OR Ceritinib OR Alectinib OR Ensartinib OR Brigatinib OR Iruplinalkib OR Envonalkib OR Lorlatinib OR “NVL-655″)). The search period was set from 1 January 2015 to 31 December 2024. To ensure the standardization and accuracy of the results, only English-language publications were included, and only articles categorized as“Original Research”or“Review”were selected. The search process is illustrated in Figure 1.

ClinicalTrials.gov is an authoritative international clinical trial registry maintained by the U.S. National Library of Medicine and the U.S. Food and Drug Administration, and was therefore used in this study for the retrieval and analysis of clinical trial data. The search criteria included: Disease Condition set to NSCLC; Intervention/Treatment specified as ALK-TKIs; and Study Type limited to Interventional studies, with a study start date range from 1 January 2015 to 31 December 2024.

### 2.2 Data analysis and visualization

Using the Full Record and Cited References from articles retrieved from the Web of Science Core Collection, information on countries/regions, institutions, authors, journals, keywords, and references was extracted for analysis. ClinicalTrials.gov was used to retrieve and analyze data on clinical trial progress. Excel was used for statistical analysis and visualization, while the Bibliometrix package (version 3.2.1) in R was applied for bibliometric data processing (Aria and Cuccurullo, 2017). CiteSpace (version 6.3. R1) and VOSviewer (version 1.6.18) were used for bibliometric mapping and visualization (van Eck and Waltman, 2010; Chen, 2020). Journal impact was evaluated using Impact Factor (IF) and 2023 Journal Citation Reports (JCR) quartiles. Centrality was calculated using CiteSpace to assess the importance and connectivity of nodes in the collaboration network. Betweenness centrality, one common measure, reflects the likelihood that a node lies on the shortest paths between other nodes. Nodes with high betweenness centrality often bridge distinct clusters or subnetworks, serving as key connectors. In CiteSpace, such nodes are highlighted with a purple ring.

## 3 Results

### 3.1 Annual publication volume and trends

**FIGURE 2:**
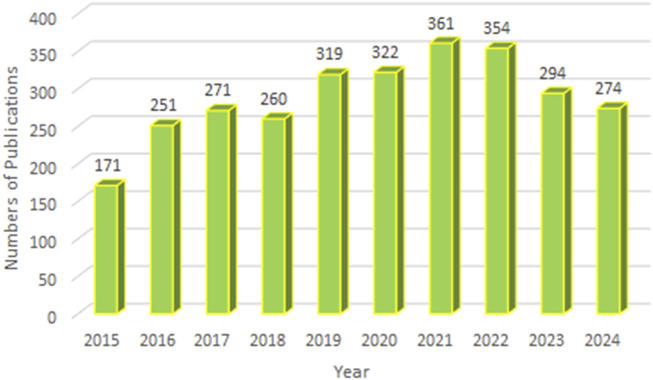
Annual publication volume and temporal trend of studies on ALK-TKIs in Non-Small Cell Lung Cancer.

A total of 2,877 publications were included in this study, comprising 2,330 original research articles and 547 reviews. As shown in Figure 2, the annual number of publications related to ALK-TKIs in NSCLC has remained consistently high over the past decade, with over 250 articles published annually since 2016, indicating sustained research interest and highlighting it as a prominent area in cancer research.

### 3.2 Countries/regions analysis

**FIGURE 3:**
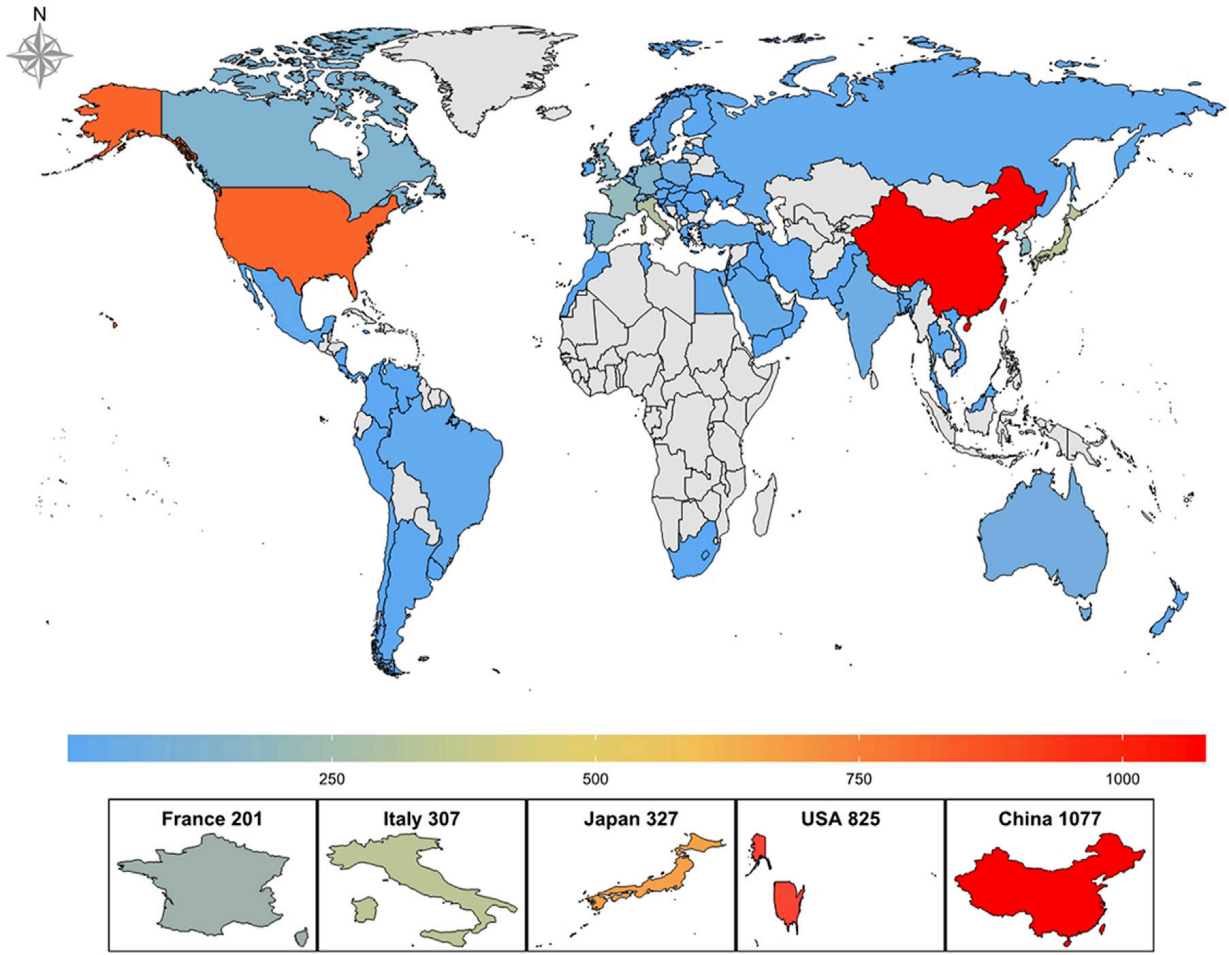
Global geographical distribution of publications on ALK-TKIs in Non-Small Cell Lung Cancer.

**FIGURE 4:**
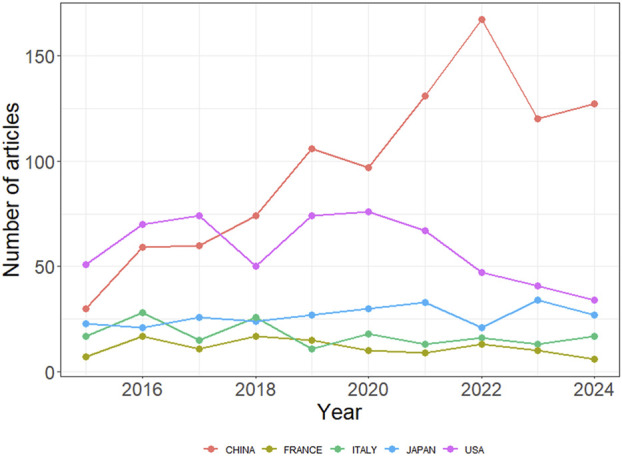
Publication trends of the top five most productive countries in ALK-TKI research for NSCLC.

**FIGURE 5:**
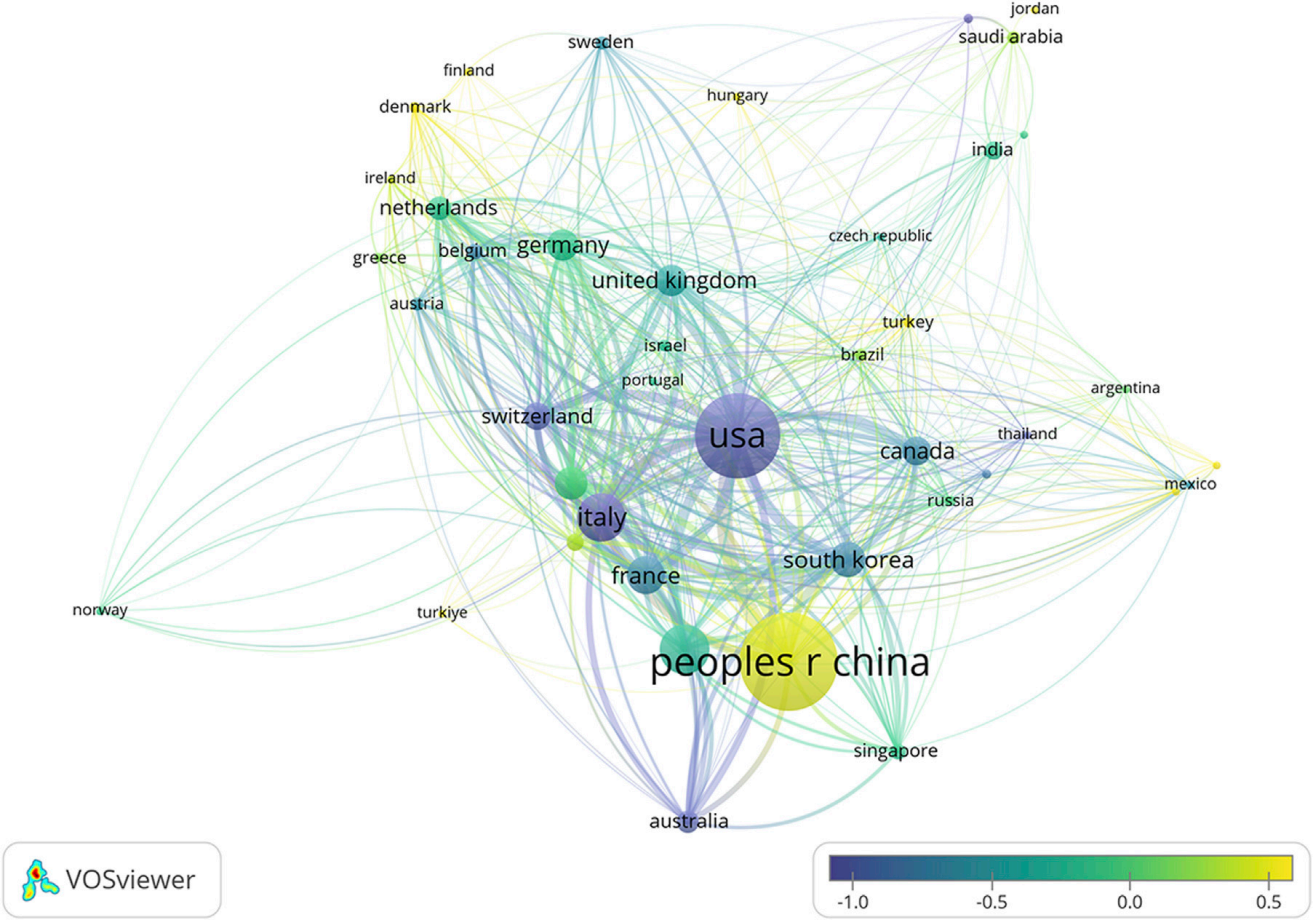
Co-authorship network of countries/regions involved in ALK-TKI research for Non-Small Cell Lung Cancer. Node size indicates the number of publications, connecting lines represent collaborative relationships, and node color reflects the average publication year.

The 2,877 papers included in this study originated from 73 countries or regions. Table 1 presents the top ten countries or regions by publication volume. Over the past decade, China led with 1,077 publications, followed by the United States (825), Japan (327), Italy (307), and France (201). Figure 3 illustrates the global geographic distribution of publications, with the top five countries—China, the United States, Japan, Italy, and France—individually labeled. Figure 4 presents the publication trends of these five countries over the past decade. Since 2017, China has surpassed the United States to become the leading contributor, maintaining a high level of output thereafter. The United States has experienced a decline in output since 2020, while the other three countries have demonstrated relatively stable trends, consistent with their overall rankings. Figure 5 displays the international collaboration network, revealing that the United States serves as a central hub of international cooperation. Recent contributions also originate from countries such as Denmark and China.

**TABLE 1:** Top 10 productive countries/regions and top 10 productive institutions related to ALK-TKI in the treatment of Non-small cell lung cancer.

Rank	Country/region	Count	Percentage (N/2,887)	Rank	Institution	Count	Location
1	China	1,077	37.43%	1	Massachusetts General Hospital	118	United States of America
2	United States of America	825	28.68%	2	University of Colorado	93	United States of America
3	Japan	327	11.37%	3	University of California, Irvine	82	United States of America
4	Italy	307	10.67%	4	National Cancer Center Japan	79	Japan
5	France	201	6.99%	5	Memorial Sloan-Kettering Cancer Center	69	United States of America
6	South Korea	181	6.29%	6	Shanghai Jiao Tong University	66	China
7	England	161	5.60%	7	Zhejiang University	60	China
8	Spain	159	5.53%	8	Harvard Medical School	57	United States of America
9	Germany	148	5.14%	9	Sun Yat-Sen University	53	China
10	Canada	131	4.55%	10	Sichuan University	52	China

### 3.3 Institutional analysis

**FIGURE 6:**
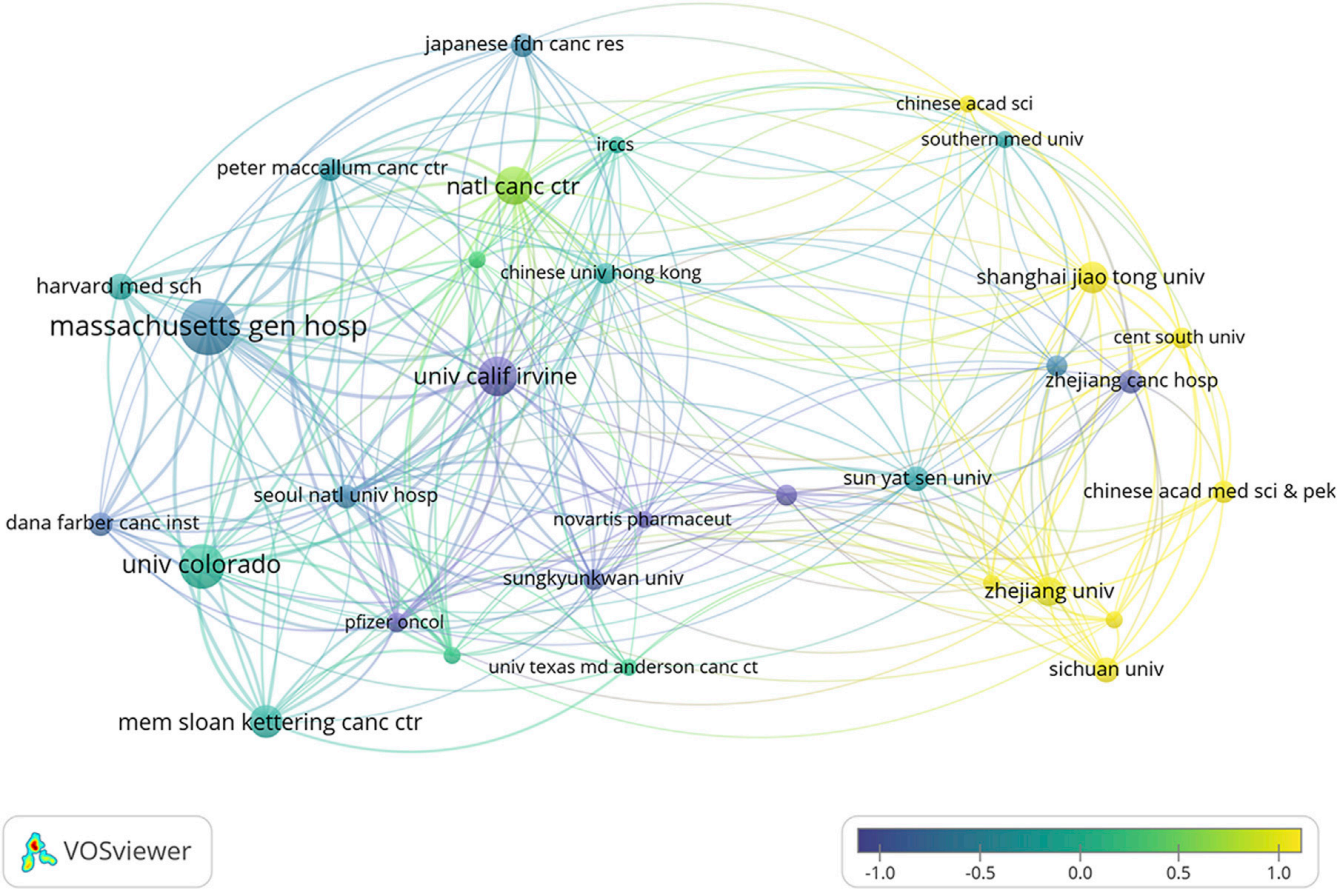
Overlay visualization of institutional co-authorship networks and average publication time in ALK-TKI research for Non-Small Cell Lung Cancer.

Over the past decade, a total of 12,010 research institutions have contributed to studies on ALK-TKIs in NSCLC. The top contributing institutions included Massachusetts General Hospital (118 publications), University of Colorado (93), University of California, Irvine (82), National Cancer Center Japan (79), and Memorial Sloan Kettering Cancer Center (69). Among the top ten institutions, the majority are based in the United States, followed by China, highlighting the leading roles of these two countries in this research domain (see Table 1 for details). Figure 6 illustrates the institutional collaboration network, with recent research contributions primarily originating from institutions such as Shanghai Jiao Tong University and Zhejiang University.

### 3.4 Journal analysis

The publications analyzed in this study were distributed across 508 academic journals (Table 2). The journal Lung Cancer published the highest number of relevant studies (185 publications), followed by Journal of Thoracic Oncology (122), Frontiers in Oncology (115), Clinical Lung Cancer (99), and Translational Lung Cancer Research (79). These journals are primarily focused on lung cancer and oncology. In terms of total citations, articles in Journal of Thoracic Oncology were cited the most (7,152), followed by Clinical Cancer Research (5,419) and Lung Cancer (4,429). The average number of citations per article also reflects journal impact to some extent: Clinical Cancer Research ranked highest with an average of 110.59 citations per article, followed by Journal of Thoracic Oncology (58.62), and Lung Cancer (23.94). Combining total citations and average citations, Clinical Cancer Research, Journal of Thoracic Oncology, and Lung Cancer emerge as the three most influential journals in this research domain. Notably, 90% of the top ten journals by publication volume were classified as JCR Q1 or Q2, indicating that research on ALK-TKIs in NSCLC is primarily published in high-quality journals. Most of the leading journals are based in the United States, followed by China, the United Kingdom, and Switzerland.

**TABLE 2:** Top 10 productive journals related to ALK-TKI in the treatment of Non-small cell lung cancer.

Journals	Count	Citation	Average citation	IF(2023)	JCR	Country/region
*Lung Cancer*	185.00	4,429.00	23.94	4.50	Q1	Netherlands
*Journal of Thoracic Oncology*	122.00	7,152.00	58.62	21.00	Q1	United States of America
*Frontiers in Oncology*	115.00	1,086.00	9.44	3.50	Q2	Switzerland
*Clinical Lung Cancer*	99.00	1,624.00	16.40	3.30	Q2	United States of America
*Translational Lung Cancer Research*	79.00	856.00	10.84	4.00	Q1	China
*Thoracic Cancer*	75.00	818.00	10.91	2.30	Q2	China
*Oncotargets and Therapy*	68.00	796.00	11.71	2.70	Q3	United Kingdom
*Cancers*	62.00	1,102.00	17.77	4.50	Q1	Switzerland
*Clinical Cancer Research*	49.00	5,419.00	110.59	10.40	Q1	United States of America
*Future Oncology*	37.00	281.00	7.59	3.00	Q2	United Kingdom

**FIGURE 7:**
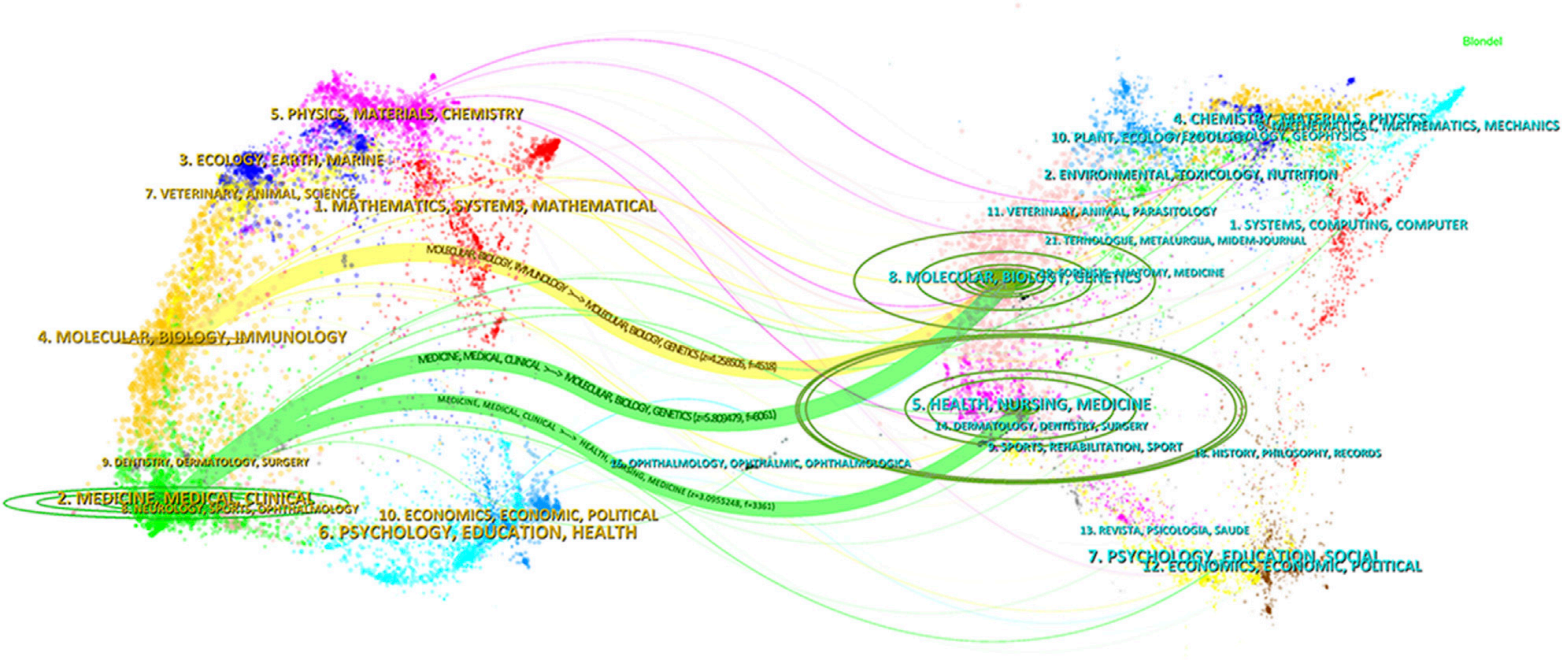
Dual-map overlay of journals related to ALK-TKIs in Non-Small Cell Lung Cancer. The citing journals are displayed on the left, and the cited journals are on the right. The curved lines represent citation paths, illustrating the disciplinary relationships between the citing and cited journals.

Figure 7 presents a dual-map overlay of journals, where citing journals are displayed on the left and cited journals on the right. The colored lines represent citation trajectories, indicating the disciplinary citation relationships between the two sides. Three prominent citation paths are visible in the figure. The citing journals are primarily concentrated in the Medicine/Medical/Clinical and Molecular/Biology/Immunology disciplines. These journals mainly cite articles published in the Molecular/Biology/Genetics and Health/Nursing/Medicine fields. This indicates that the research on ALK-TKIs in NSCLC is largely grounded in molecular and biological science, with substantial integration into clinical medicine and health-related research domains.

### 3.5 Author and reference analysis

**FIGURE 8:**
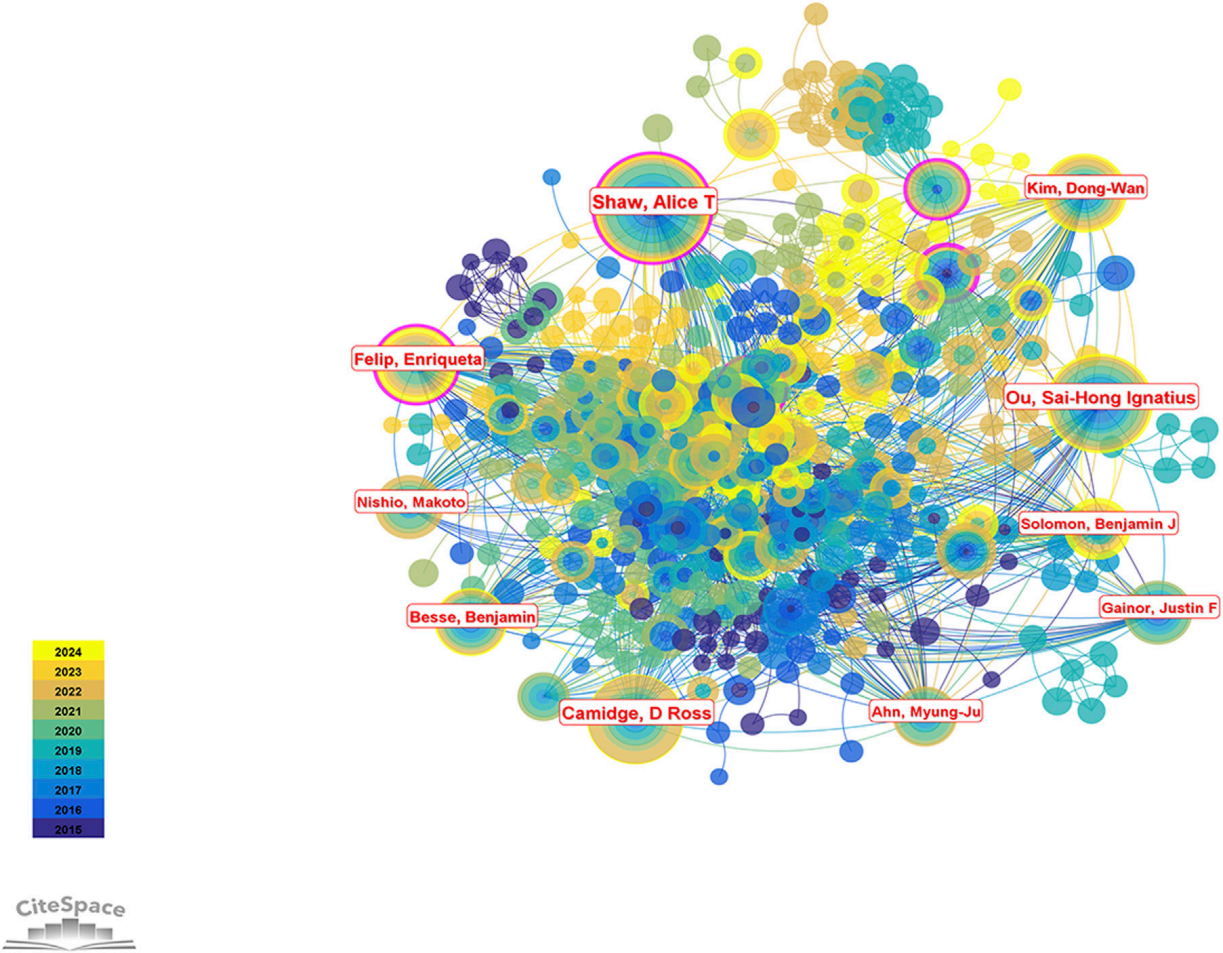
Author collaboration network map in the field of ALK-TKI research for NSCLC. Nodes represent individual authors, and links represent co-authorship relationships. Purple rings around the nodes indicate authors with high betweenness centrality.

A total of 16,078 authors contributed to research on ALK-TKIs in the field of NSCLC over the past decade. The top five most prolific authors were Alice T. Shaw (76 publications), Sai-Hong Ignatius Ou (66), D. Ross Camidge (56), Enriqueta Felip (50), and Dong-Wan Kim (Soo et al., 2023) (see Table 3 for the top ten authors). In the author collaboration network (Figure 8), purple circles surrounding the nodes for Alice T. Shaw and Enriqueta Felip indicate a centrality score greater than 0.1. As shown in Table 4, both authors had a centrality of 0.13—the highest among all authors—highlighting their pivotal influence in this research area.

**TABLE 3:** Top 10 productive authors related to ALK-TKI in the treatment of Non-small cell lung cancer.

Rank	Author	Count	Centrality
1	Alice T. Shaw	76	0.13
2	Sai-Hong Ignatius Ou	66	0.05
3	D. Ross Camidge	56	0.04
4	Enriqueta Felip	50	0.13
5	Dong-Wan Kim	42	0.08
6	Benjamin Besse	34	0.05
7	Benjamin Solomon	32	0.06
8	Justin Gainor	30	0.02
9	Myung-Ju Ahn	28	0.07
10	Makoto Nishio	28	0.04

**TABLE 4:** Top 10 high-cited references related to ALK-TKI in the treatment of Non-small cell lung cancer.

Rank	Reference	First author	Citation	Centrality	Journal
1	First-line crizotinib versus chemotherapy in ALK-positive lung cancer	Benjamin Solomon	558	0.01	New England Journal of Medicine
2	Alectinib versus crizotinib in Untreated ALK-positive non-small-cell lung cancer	Solange Peters	534	0.2	New England Journal of Medicine
3	Crizotinib versus chemotherapy in advanced ALK-positive lung cancer	Alice T. Shaw	468	0.01	New England Journal of Medicine
4	First-line ceritinib versus platinum-based chemotherapy in advanced ALK-rearranged non-small-cell lung cancer (ASCEND-4): a randomised, open-label, phase 3 study	Jean-Charles Soria	346	0.06	Lancet
5	Ceritinib in ALK-rearranged non-small-cell lung cancer	Alice T. Shaw	291	0.09	New England Journal of Medicine
6	Alectinib versus crizotinib in patients with ALK-positive non-small-cell lung cancer (J-ALEX): an open-label, randomised phase 3 trial	Toyoaki Hida	284	0.02	Lancet
7	Brigatinib versus Crizotinib in ALK-Positive non-small-cell lung cancer	D Ross Camidge	273	0.04	New England Journal of Medicine
8	First-line lorlatinib or crizotinib in advanced ALK-positive lung cancer	Alice T. Shaw	255	0.03	New England Journal of Medicine
9	Crizotinib in ROS1-rearranged non-small-cell lung cancer	Alice T. Shaw	254	0.03	New England Journal of Medicine
10	Alectinib in crizotinib-refractory ALK-rearranged non-small-cell lung cancer: a phase ii global study	Sai-Hong Ignatius Ou	233	0.04	Journal of Thoracic Oncology

**FIGURE 9:**
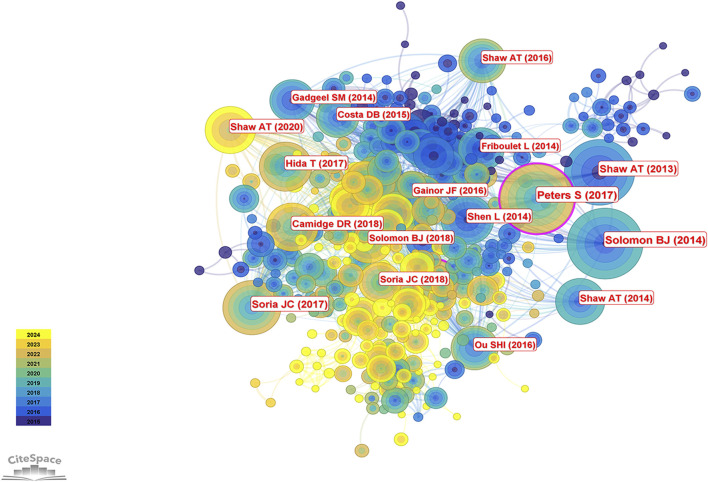
Co-citation network map of references in ALK-TKI research for NSCLC.

The 2,877 articles included in this study collectively cited 39,574 references. According to Table 5, the most frequently cited reference was by Solomon et al. (2014), cited 558 times, followed by Peters et al. (2017) with 534 citations and the highest centrality. The third most cited study was authored by Shaw et al. (2013), cited 468 times; this was followed by studies by Soria et al. (2017) (346 citations) and another by Shaw et al. (2014). Taken together, Alice T. Shaw not only ranks first in publication volume but also has four studies among the ten most cited references, underscoring her significant contributions and leadership in the field of ALK-TKI therapy for NSCLC. Figure 9 presents the co-citation network of the most influential references.

**TABLE 5:** Comparison of efficacy and clinical endpoints of major ALK-TKIs in NSCLC.

Intervention	Generation	Key trial	Median PFS (months)	Median OS (months)	PFS HR	OS HR	CNS ORR	Grade ≥3 AEs
Crizotinib vs. chemotherapy	1st	PROFILE 1014	10.90	NR	0.45	0.76	18.00%	50.30%
Alectinib vs. crizotinib	2nd	ALEX	34.80	NR	0.43	0.67	81.00%	52.00%
Brigatinib vs. crizotinib	2nd	ALTA-1L	24.00	NR	0.48	0.81	78.00%	70.00%
Ensartinib vs. crizotinib	2nd	eXalt3	25.80	NR	0.51	0.91	64.00%	44.80%
Lorlatinib vs. crizotinib	3rd	CROWN	NR	Not reported	0.19	Not reported	83.00%	77.00%

Abbreviations: PFS, progression-free survival; OS, overall survival; HR, hazard ratio; ORR, objective response rate; AEs, adverse events; NR, not reached.

### 3.6 Keywords analysis

**FIGURE 10:**
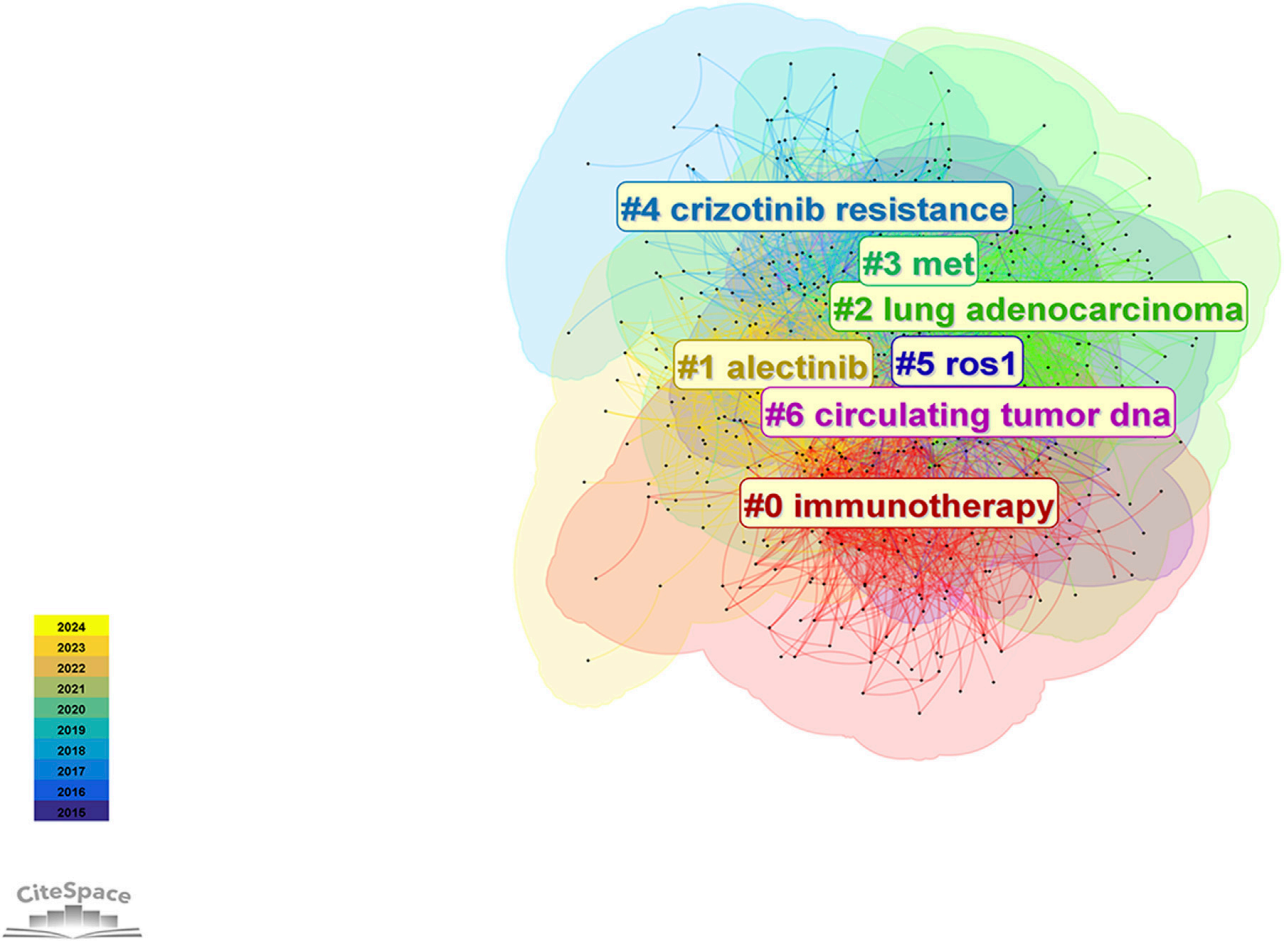
Keyword clustering and co-occurrence network map in ALK-TKI research for NSCLC.

A total of 3,609 keywords were identified in research related to ALK-TKIs in NSCLC over the past decade. Cluster analysis (Figure 10) grouped these keywords into seven distinct clusters: #0 immunotherapy, #1 alectinib, #2 lung adenocarcinoma, #3 MET, #4 crizotinib resistance, #5 ROS1, and #6 circulating tumor DNA (ctDNA). These clusters primarily encompass topics related to ALK-TKIs (#1 alectinib, #4 crizotinib resistance), including mechanisms of drug resistance. Clusters #3 (MET) and #5 (ROS1) involve gene alterations associated with ALK mutations; notably, MET gene amplification is a common mechanism of NSCLC drug resistance, while ROS1 shares significant structural and therapeutic similarities with ALK. Cluster #0 (immunotherapy) reflects efforts to apply immune checkpoint inhibitor therapy in ALK-positive NSCLC patients. Finally, cluster #6 (circulating tumor DNA) highlights the growing interest in liquid biopsy techniques for prognosis prediction and dynamic monitoring of gene mutation status in ALK-positive NSCLC during the last decade.

**FIGURE 11:**
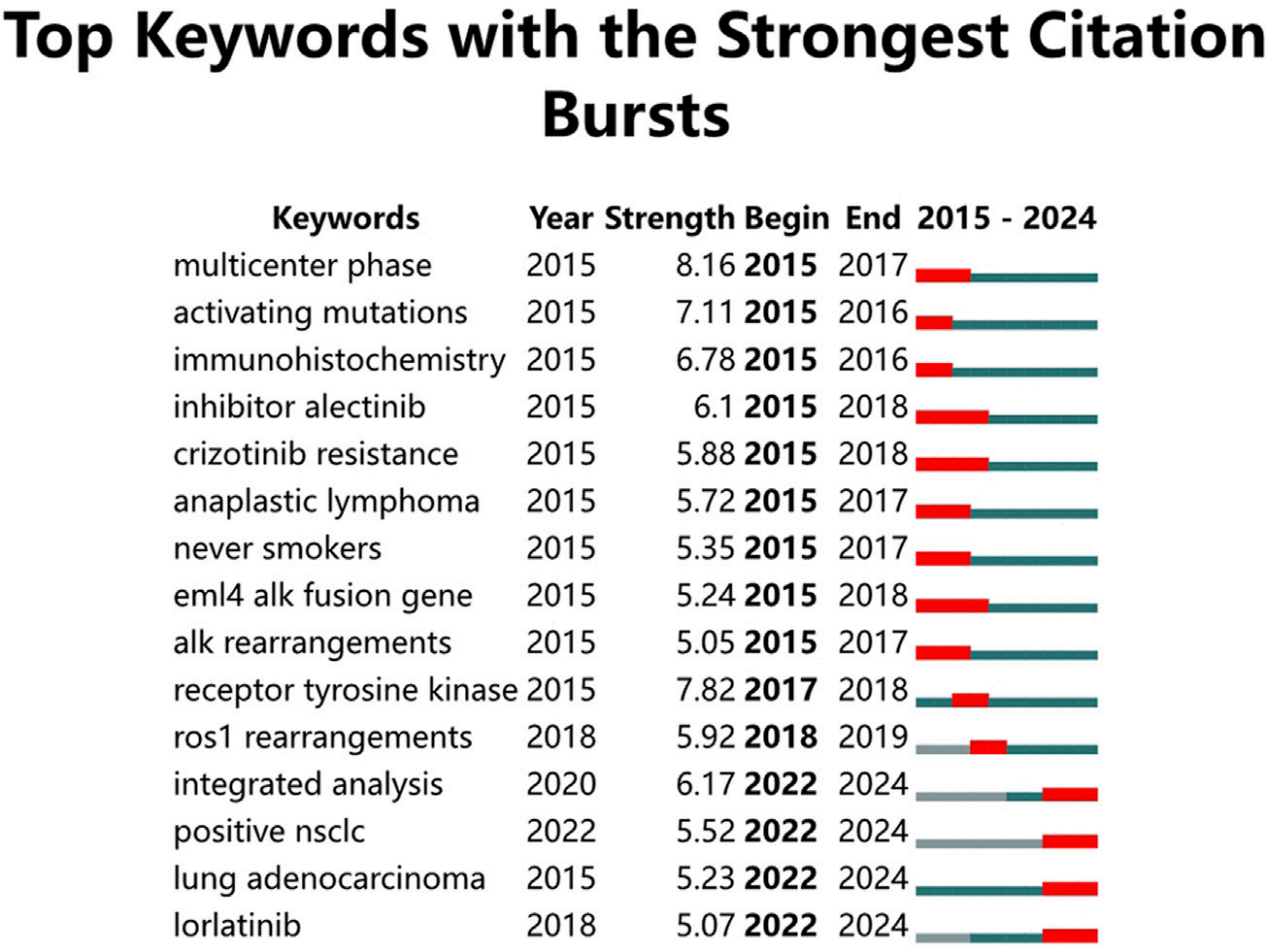
Keyword burst detection map in ALK-TKI research for NSCLC.

The keyword burst detection map (Figure 11) highlights the 15 keywords with the strongest citation bursts over the past decade. In the early stage of this research field, starting from 2015, the primary focus was on ALK mutation types such as “EML4-ALK fusion gene” and “ALK rearrangements”, as well as on ALK-TKIs. Research initially concentrated on the therapeutic effects and resistance mechanisms of the first-generation ALK-TKI crizotinib (“crizotinib resistance”) and the second-generation ALK-TKI alectinib. From 2022 onwards, keywords such as “integrated analysis”, “positive NSCLC”, “lung adenocarcinoma”, and “lorlatinib” have emerged as new hotspots, indicating that future research will focus on ALK-positive NSCLC, particularly lung adenocarcinoma. Moreover, lorlatinib, a third-generation ALK-TKI, represents a promising area with substantial potential for ongoing investigation.

### 3.7 Clinical trial analysis

**FIGURE 12:**
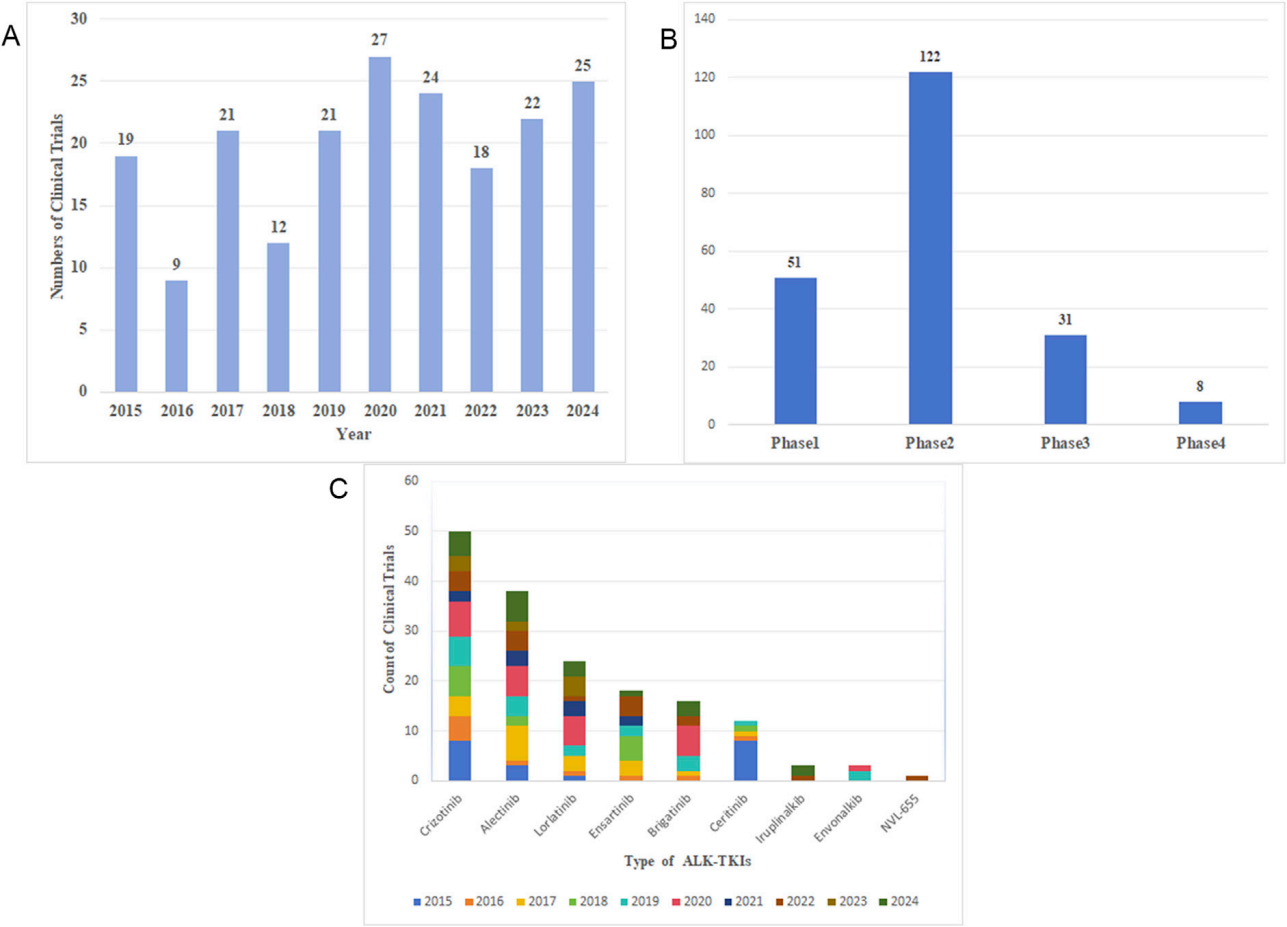
**(A)** Annual number of clinical trial publications. **(B)** Clinical trial phases distribution. **(C)** Number of clinical trials and initiation year for each ALK-TKI agent.

Over the past decade, 198 clinical trials related to ALK-TKIs for NSCLC have been registered on ClinicalTrials.gov. The continuous progress in clinical trials has been fundamental to advancing research in this field and improving patient prognosis. The annual number of study initiations has fluctuated over the last 10 years, with the past 5 years consistently exceeding 20 new trials per year (Figure 12A). As shown in Figure 12B, most registered trials are currently in Phase 2, followed by Phase 1, while Phase 4 trials are the least common. This distribution reflects the current stage of research development, indicating a need for more early-phase trials before progressing to later phases, thereby enriching clinical therapeutic options in the future. Furthermore, the distribution of clinical trials by ALK-TKI type over time (Figure 12C) reveals that crizotinib-related trials are the most numerous, followed by those involving alectinib and lorlatinib, representing the first-, second-, and third-generation ALK-TKIs, respectively. Notably, the number of new trials initiating in 2024 remains high, demonstrating sustained research interest in these agents. Although only a limited number of clinical trials have been conducted on fourth-generation ALK-TKIs such as NVL-655, this remains a promising and emerging area of research.

### 3.8 Comparative efficacy and key clinical endpoints of ALK-TKIs in NSCLC

As mentioned in the introduction, the treatment paradigm for ALK-positive NSCLC has evolved considerably due to the interplay between clinical demands and advances in molecular oncology, resulting in successive generations of ALK-TKIs. Multiple pivotal clinical trials have assessed the efficacy, safety, and survival benefits of these agents, as summarized in Table 5.

Crizotinib, the first-generation ALK-TKI, demonstrated significantly improved PFS over standard chemotherapy in the PROFILE 1014 study (median PFS: 10.9 vs. 7.0 months). In the final analysis of overall survival (OS), the crizotinib arm had not reached (NR) median OS, whereas the chemotherapy arm reported a median OS of 47.5 months, suggesting a survival benefit with crizotinib (Solomon et al., 2018). Nevertheless, crizotinib’s limited central nervous system (CNS) penetration restricts its efficacy in patients with brain metastases.

To address the challenge of acquired resistance and improve control of CNS metastases, second-generation ALK inhibitors such as alectinib and brigatinib were developed. The ALEX trial demonstrated the superiority of alectinib over crizotinib as first-line therapy, with a median progression-free survival (PFS) of 34.8 months versus 10.9 months, respectively. Moreover, alectinib significantly reduced the cumulative incidence of CNS progression (Peters et al., 2017). Final OS data from the ALEX trial showed that the median OS had not been reached in the alectinib group, compared to 57.4 months in the crizotinib arm, highlighting a durable survival benefit (Mok et al., 2020).

Similarly, the ALTA-1L study demonstrated that brigatinib conferred a median PFS of 24.0 months, significantly longer than the 11.0 months observed with crizotinib. Brigatinib also exhibited superior intracranial efficacy and a favorable safety profile (Camidge et al., 2021). Another second-generation ALK-TKI, ensartinib, has also demonstrated superior therapeutic efficacy compared to crizotinib. In the eXalt3 trial, ensartinib achieved a median PFS of 25.8 months, significantly longer than 12.7 months with crizotinib, along with improved CNS activity and a favorable safety profile (Horn et al., 2021).

Lorlatinib, a third-generation ALK inhibitor, was specifically developed to overcome resistance mutations and improve CNS penetration. In the CROWN study, the median PFS for lorlatinib was not reached at the time of the primary analysis, while the crizotinib group reported a median PFS of 9.3 months. Notably, lorlatinib achieved an intracranial objective response rate (iORR) of 83% in patients with measurable brain metastases, highlighting its potent intracranial activity (Solomon et al., 2024).

Across key clinical trials of ALK-TKIs, the primary efficacy endpoints include PFS, OS, objective response rate (ORR), and the incidence of grade ≥3 adverse events. While newer-generation ALK-TKIs provide superior intracranial control and broader coverage of resistance mutations, the optimal therapeutic strategy should be personalized based on a comprehensive assessment of drug characteristics, safety profiles, and individual patient tolerance.

## 4 Discussion

The present study provides a comprehensive bibliometric analysis of research on ALK-TKIs for NSCLC between 2015 and 2024, alongside an analysis of clinical studies conducted over the past decade. A total of 2,877 publications and 198 clinical trials were identified, indicating that this field remains a research hotspot. Although annual publications peaked at 361 in 2021 and have since shown a slight decline, research output remains substantial with 274 studies projected for 2024. Additionally, the number of clinical trials registered in 2024 reached 25, demonstrating a gradual increase since 2022. These trends collectively highlight the sustained and growing potential of this research area.

The studies included in this analysis originated from 73 countries/regions, underscoring the global scope of research on ALK-TKIs in NSCLC. China not only contributed the largest number of publications but also demonstrated the most pronounced growth trend among the top publishing countries. This can be attributed to both the rapid advancement in China’s research capabilities and its large population of ALK-positive NSCLC patients, given the high prevalence of lung cancer in the country (Han et al., 2024; Gou and Wu, 2014; Yang et al., 2016). In terms of international collaboration, the United States—ranked second in publication volume—exhibited the highest level of global cooperation. By contrast, countries such as China had relatively fewer international research exchanges. This discrepancy may be partly explained by regional differences in disease incidence and preferred treatment protocols for ALK-positive NSCLC. Going forward, enhanced collaboration among countries/regions with similar epidemiological patterns and clinical practices should be encouraged. Regarding research institutions, most of the high-output centers are located in the United States and China. Notably, a significant proportion of recent publications originated from China, particularly from institutions such as Shanghai Jiao Tong University. This suggests that ongoing developments from top research centers in both countries warrant close attention from scholars in this field.

Studies on ALK-TKIs for NSCLC are predominantly published in journals such as Lung Cancer, Journal of Thoracic Oncology, and Frontiers in Oncology. These journals provide an overview of mainstream research activity in the field. For high-impact findings, particular attention should be paid to studies published in Journal of Thoracic Oncology, Clinical Cancer Research, and Lung Cancer. The journal dual-map overlay indicates that the foundational research in this area primarily stems from the fields of molecular biology and genetics, while the target publication venues are concentrated in clinical and medical disciplines. This trend highlights the translational nature of ALK-TKI research for NSCLC, emphasizing the progression from basic science to clinical application.

From an author perspective, when both publication count and citation frequency are considered as key indicators, Alice T. Shaw emerges as the most influential contributor in the field. As highlighted in the results, her centrality exceeds 0.1, further underscoring her pivotal role in ALK-TKI research for NSCLC. In addition to Shaw, three other scholars—Sai-Hong Ignatius Ou, D. Ross Camidge, and Benjamin Solomon—also ranked among the top ten in both publication volume and citation frequency. Notably, the most frequently cited reference in this field is authored by Benjamin Solomon. For researchers seeking to understand authoritative findings and evolving trends in this area, the work of Alice T. Shaw, Benjamin Solomon, Sai-Hong Ignatius Ou, and D. Ross Camidge warrants close attention.

Keywords serve as a concise summary of research content. In the field of ALK-TKIs for NSCLC, in addition to terms such as “ALK-TKI” and “lung adenocarcinoma,” the prominent keywords are primarily associated with three major research areas: drug resistance mechanisms, circulating tumor DNA, and immunotherapy. Although the development and clinical application of successive generations of ALK-TKIs have significantly improved outcomes for patients with ALK-positive NSCLC, drug resistance remains a major challenge (Poei et al., 2024). Resistance to ALK-TKIs can be broadly classified into primary and secondary resistance. Primary resistance is relatively rare and typically reported in case studies (Smolle et al., 2021; Siblini et al., 2023). This study focuses on secondary resistance, particularly ALK-dependent mechanisms. ALK-dependent resistance primarily involves mutations within the ALK kinase domain and amplification of ALK gene copy number. Mutations in the kinase domain alter the ATP-binding site or protein conformation, thereby impairing TKI binding and reactivating downstream signaling pathways (Schneider et al., 2023). For example, in crizotinib-resistant patients, common mutations include L1196M, G1269A, and G1202R ((Patcas et al., 2022)). Notably, G1202R is also the most frequent mutation associated with resistance to second-generation ALK-TKIs such as alectinib and ceritinib (Lin et al., 2016; Shen et al., 2022). Some resistant mutations can be overcome by switching to alternative ALK-TKIs. For instance, ensartinib is effective against I1171, F1174, V118L, and C1156Y mutations—key mechanisms of resistance to alectinib and ceritinib (Xia et al., 2025). Lorlatinib, a third-generation ALK-TKI, can overcome multiple kinase domain mutations, including G1202R and I1171 N/S/T (Shiba-Ishii et al., 2022). Interestingly, the I1171N mutation may re-sensitize patients to alectinib after lorlatinib resistance (Okada et al., 2019). However, most currently approved ALK-TKIs are ineffective against compound mutations involving G1202R. In contrast, fourth-generation ALK-TKIs such as NVL-655 have demonstrated preclinical efficacy in targeting these resistant mutations (Lin et al., 2024). Given the dynamic evolution of resistance mutations in ALK-positive NSCLC patients during treatment, conventional tissue-based genetic testing may be insufficient due to its lag time. Liquid biopsy, particularly the analysis of circulating tumor DNA (ctDNA), has gained clinical relevance as a non-invasive method for real-time monitoring of resistance mutations (Hua et al., 2022). Moreover, ctDNA levels have been proposed as potential biomarkers for predicting treatment response and prognosis in patients receiving ALK-TKIs (Soo et al., 2023).

In the keyword analysis, immunotherapy emerged as one of the prominent clusters, reflecting increasing interest in combining ALK-TKIs with other therapeutic modalities. However, current evidence suggests that the combination of ALK-TKIs with immune checkpoint inhibitors (ICIs) has limited clinical utility and raises safety concerns in ALK-positive NSCLC. The CheckMate 370 trial (Spigel et al., 2018), which investigated first-line nivolumab in combination with crizotinib, was terminated prematurely due to severe hepatotoxicity observed in 38% of patients, including treatment-related deaths. Similarly, another clinical study evaluating ICIs combined with crizotinib reported significantly increased hepatotoxicity (Lin et al., 2019). These findings consistently indicate that the direct combination of ICIs and ALK-TKIs may not be a viable therapeutic strategy. The IMpower150 trial (Socinski et al., 2021), although not exclusively focused on patients receiving ALK-TKIs, offers insight into immunotherapy in ALK-positive NSCLC. In this trial, patients with EGFR or ALK-positive NSCLC who were chemotherapy-naïve or had failed/intolerant prior TKI therapy received atezolizumab + bevacizumab + carboplatin + paclitaxel (ABCP) versus bevacizumab + carboplatin + paclitaxel (BCP). The ABCP regimen demonstrated superior efficacy, with a median progression-free survival (PFS) of 10.2 months vs. 6.9 months (HR = 0.60), and median OS of 29.4 months vs. 18.1 months (HR = 0.60). These results suggest that combining immunotherapy with anti-angiogenic agents and chemotherapy may be a more effective and tolerable treatment approach for advanced ALK-positive NSCLC. In addition to ICIs, future directions in immunotherapy for ALK-positive NSCLC may include the development of ALK-specific cancer vaccines, which could be used in combination with ALK-TKIs or ICIs to enhance antitumor efficacy against ALK-rearranged NSCLC (Voena et al., 2015). Another promising approach is oncolytic virotherapy, which aims to selectively lyse ALK-rearranged tumor cells, thereby releasing tumor-associated antigens and activating immune responses within the tumor microenvironment (Ma et al., 2023). These strategies, along with other rational combination regimens, are being explored to enhance tumor immunogenicity and overcome the inherently “cold” immune landscape characteristic of ALK-rearranged NSCLC.

The mutational landscape of ALK-positive NSCLC is often highly complex, and resistance to existing ALK-TKIs remains a major therapeutic challenge. This issue necessitates further investigation into the sequential administration of different generations of ALK-TKIs, the integration of combination therapies, and the development of next-generation ALK inhibitors with improved efficacy and resistance profiles.

In the field of ALK-TKIs for NSCLC, clinical research remains a top priority. As evidenced by the references, nearly all of the ten most cited studies are clinical trials evaluating the efficacy and safety of ALK inhibitors. An analysis of clinical trial activity over the past decade reveals sustained research intensity, with a predominant focus on agents that have been clinically available for a longer period. The majority of trials are early-phase (Phase 1 and 2) studies, reflecting the current stage of development and the clinical complexities of ALK-positive NSCLC, such as high rates of drug resistance and substantial interpatient variability. High-quality, large-scale clinical trials are therefore essential to generate robust evidence for guiding therapeutic decision-making. Looking forward, more rigorous clinical research is anticipated, particularly the advancement of next-generation ALK inhibitors—such as NVL-655—into later-phase trials, which may further optimize treatment outcomes for this molecular subtype of NSCLC.

## 5 Limitation

Although this study systematically compiled and analyzed a decade of research on ALK-TKIs for NSCLC from a bibliometric perspective, several limitations inherent to bibliometric analyses should be acknowledged. First, the data sources were limited to the Web of Science Core Collection and ClinicalTrials.gov databases. While the Web of Science provides comprehensive bibliographic records including full metadata and cited references essential for bibliometric studies, and ClinicalTrials.gov offers extensive clinical trial data, some relevant publications indexed exclusively in other databases such as Scopus may have been inadvertently omitted. Second, the bibliometric tools employed in this study (CiteSpace, VOSviewer, and the R platform) are primarily optimized for English-language literature. To ensure accuracy and scientific rigor, only articles published in English were included, potentially excluding relevant non-English studies. Third, this analysis focused on original research articles and reviews, which best represent the development of the field; grey literature (e.g., conference proceedings, editorials, case reports) was excluded to improve data quality and consistency. Lastly, due to the inherent time lag in citation accrual, the impact of more recently published studies may be underestimated.

## 6 Conclusion

This study systematically summarizes and analyzes the research history, current status, hotspots, and future trends of ALK-TKIs in the treatment of NSCLC over the past decade from a bibliometric perspective. Research in this field remains highly active and rapidly evolving. Despite the availability of numerous effective ALK-TKIs in clinical practice, the issue of drug resistance in ALK-positive NSCLC patients persists. Therefore, the ongoing development of both established and novel drugs, investigation of the safety and efficacy of various therapeutic strategies, and identification of more accurate predictive biomarkers for relapse, resistance mutations, and prognosis remain urgent priorities. Furthermore, there is a critical need for updated, high-quality randomized clinical trials to generate robust evidence that can guide optimal clinical management. With continuous scientific advancement, it is anticipated that personalized, precise, and effective treatment approaches for ALK-positive NSCLC patients will be realized in the near future.

## Data Availability

The datasets presented in this study can be found in online repositories. The names of the repository/repositories and accession number(s) can be found in the article/supplementary material.
